# Persistence and change in behavioural problems during early childhood

**DOI:** 10.1186/s12887-019-1631-3

**Published:** 2019-07-26

**Authors:** Stephanie D’Souza, Lisa Underwood, Elizabeth R. Peterson, Susan M. B. Morton, Karen E. Waldie

**Affiliations:** 1 School of Psychology, University of Auckland, Auckland, New Zealand; 2 Centre for Longitudinal Research - He Ara ki Mua, University of Auckland, Private Bag 92019, Auckland, New Zealand; 3 Centre of Methods and Policy Application in the Social Sciences, University of Auckland, Auckland, New Zealand; 4 Department of Psychological Medicine, University of Auckland, Private Bag 92019, Auckland, New Zealand; 5 School of Population Health, University of Auckland, Auckland, New Zealand

**Keywords:** SDQ, Child behaviour, Cohort, Longitudinal, Early childhood

## Abstract

**Background:**

Behavioural problems and psychopathology can present from as early as the preschool period. However there is evidence that behavioural difficulties may not be stable over this period. Therefore, the current study was interested in evaluating the persistence and change in clinically relevant behavioural problems during early childhood in a population-based New Zealand birth cohort.

**Methods:**

Behaviour was assessed in 5896 children when they were aged 2 and 4.5 years using the Strengths and Difficulties Questionnaire (SDQ). Correlations and mean differences in subscale and total difficulties scores were examined. Scores were then dichotomised into normal/borderline and abnormal ranges to evaluate the persistence and change in significant behavioural problems. Chi-square analyses and ANOVAs were used to determine the association between sociodemographic and birth variables, and preschool behavioural stability.

**Results:**

Raw scores at ages 2 and 4.5 years were moderately correlated, with most measures showing a small but significant decrease in mean scores over time. The majority of children who showed abnormal behaviour at 2 years improved at 4.5 years (57.9% for total difficulties). However, a notable proportion persisted in their difficulties from 2 to 4.5 years (42.1% for total difficulties). There was a small percentage of children who were categorised as abnormal only at 4.5 years. Children with difficulties at one or both time points had a greater proportion who were the result of an unplanned pregnancy, lived in highly deprived urban areas, and had mothers who were younger, of Māori and Pacific ethnicity and were less educated.

**Conclusions:**

Not all children who show early behavioural difficulties persist in these difficulties. Those whose difficulties persist were more likely to experience risk factors for vulnerability relative to children with no difficulties. Results suggest that repeated screening for early childhood behavioural difficulties is important.

**Electronic supplementary material:**

The online version of this article (10.1186/s12887-019-1631-3) contains supplementary material, which is available to authorized users.

## Background

Clinically significant psychiatric disorders can present as early as preschool age, with the following rates reported in children aged 2 to 5 years: 2 to 5.7% for ADHD; 4 to 16.8% for ODD, 0 to 4.6% for CD; 0 to 2.1% for depression; 0.3 to 9.4% for anxiety disorder [1]. Within New Zealand, it is estimated that approximately 10% of children aged 2 to 4 years show clinically significant total behavioural difficulties [2, 3]. Furthermore, there is evidence that behavioural difficulties identified in children can persist and increase a child’s risk of later adverse outcomes. For example, children who show behavioural problems during childhood are at an increased risk of ongoing mental health difficulties [4–8], a greater physical health burden [8], relationship and parenting problems [4, 9], poor academic outcomes [10], criminal behaviour [4, 11], substance abuse [4, 12], as well as teen pregnancy and sexual risk-taking [4, 13]. These studies typically focus their initial assessments on children around school age or older. However, it has recently been demonstrated that difficulties that persist throughout childhood can be measured in children from as early as their second year of life [14, 15].

The studies mentioned above illustrate a shift from viewing clinically significant behavioural problems as distinct episodes to considering them as recurrent or persistent issues instead. Existing research typically investigates behavioural stability using continuous measures [15–18]. However, few studies focusing solely on early childhood have evaluated the persistence or change in clinically significant preschool behavioural problems. Given the developmental changes that occur during early childhood, it is common to believe that problem behaviours are transient and likely to reduce as the child grows older. However, this may be preventing children with genuine behavioural difficulties from getting the assistance and intervention that is needed. It is particularly important to address these behavioural concerns during the preschool period, so that the child is well prepared and adjusted for the demands of school.

The few studies that have been conducted suggest that behavioural problems in early childhood can persist for a proportion of children. Mathieson and Sanson evaluated social, internalising and externalising behavioural problems in a Norwegian community sample when children were 18 months and 30 months [14]. As most children scored close to the norm when behaviours were evaluated continuously (using the Behaviour Checklist [19]), children were categorised as showing either problematic or non-problematic behaviour at the two time points. Children were categorised as problematic if they scored at or above 1.5 standard deviations above the mean. While 2.5–3.9% of the overall sample showed persistence in behavioural problems, the authors found that approximately 37% of children with problems at 18 months persisted in their difficulties at 30 months. When looking at the association between the continuous measures of behaviour at each time point, the authors found moderate correlations.

A separate study by Briggs-Gowan, Carter, Bosson-Heenan, Guyer and Horwitz investigated whether preschool problem behaviour persisted in children from a Conneticut birth cohort [15]. Children were 12 to 40 months when initially assessed for behavioural problems and followed up a year later when they were aged 23 to 48 months. Using the Infant-Toddler Social and Emotional Assessment to measure internalising, externalising, dysregulation and total problems, children were categorised as having behavioural problems if they scored at or above the 90th percentile. The study found that 49.9% of children persisted from time 1 to time 2 in total and externalising problems, with lower persistence rates observed for the internalising (37.8%) and dysregulation (38.7%) domains.

The studies by Mathiesen and Sanson [14] and by Briggs-Gowan et al. [15] indicate that a substantial proportion of children who initially identify as showing behavioural difficulties do improve over the early childhood period, but a notable proportion still persist in these difficulties. This suggests that repeated screening from early in childhood is important for identifying these children with persistent behavioural difficulties.

In New Zealand (NZ), health and development checks are conducted on all children registered with a primary care practitioner at several time points in early childhood, starting from birth through to when the children are 4 years [20]. The aim of the check is to identify any difficulties the child may have, so that their needs are met and they are given the opportunity for optimal development. Currently, behavioural difficulties are only assessed at the 4 year health and developmental check, known as the B4 School Check, using the Strengths and Difficulties Questionnaire (SDQ) [21]. However, if similar patterns of persistence and change in behavioural problems occur in the NZ population, it may be beneficial to also conduct behavioural screening at prior health and development checks, so that intervention can occur earlier and the needs of children with persistent difficulties are adequately addressed. It is also important to explore the characteristics of children who show different behavioural development profiles, as this will indicate whether certain sociodemographic populations are more at risk of persistent behavioural problems.

The *Growing Up in New Zealand* study is a longitudinal, prospective study consisting of a large population-based birth cohort. The study assessed child behaviour when children in the cohort were aged 2 and 4.5 years using the SDQ, the same measure that is used in the B4 School Check. The assessment of behavioural difficulties at age 2 was a unique feature of this study, as this was the first time the SDQ was administered and validated in a sample as young as 2 years [3]. While we were unable to evaluate the sensitivity and specificity of the SDQ at this age, the questionnaire showed satisfactory reliability and structural validity at 2 years [3]. Furthermore, the questionnaire is meant to be used as screening tool to identify children who likely show significant behavioural problems and are in need of further assessment, rather than as a diagnostic tool. As the SDQ showed good psychometric properties in our cohort at 2 years and has been extensively validated in children aged 4 to 12 years [22], this enables us to investigate whether persistence and change in preschool behavioural problems is also observed in a NZ population, using the same screening instrument that is formally used by NZ healthcare professionals.

Using data from the *Growing Up in New Zealand* cohort, the current study firstly aimed to evaluate whether measures of behaviour at two different time points in preschool are closely correlated, and whether there are any developmental changes in behavioural scores as children move from the early preschool period (2 years) to the late preschool period (4.5 years). We hypothesised that behavioral scores at both time points will be at least moderately correlated, but there will be a slight decrease in externalising behavior, peer problems and total difficulties, as a result of developmental changes and increased social interaction as children get older. Secondly, the study was interested in calculating the rates of persistence or change in the categorisation of behavioural difficulties during this early childhood period (i.e. 2 to 4.5 years). We hypothesised that the majority of children identified as showing behavioural difficulties at 2 years will improve at 4.5 years, but a notable proportion will persist in their difficulties. Finally, we were interested in evaluating the sociodemographic characteristics of each of the apparent behavioural development profiles.

## Methods

### Design and participants

Participants were members of the *Growing Up in New Zealand* study. Details of the study’s design and recruitment procedure can be found elsewhere [23, 24]. In brief, the study’s cohort consists of a socioeconomically and ethnically diverse sample of children, recruited via 6822 pregnant women who had expected delivery dates between 25th April 2009 and 25th March 2010. Pregnant women were recruited from a geographical area that contains approximately one third of the NZ birth population, and covers three contiguous District Health Board regions [23]. Recruited mothers were found to be comparable to NZ parents on key demographic measures, such as maternal age, ethnicity, parity and area-level deprivation [23]. Children in the study were not significantly different from national births on sex and singleton births, though fewer children in the cohort were born low birth weight or preterm [25]. However, these latter statistically significant differences reflect small absolute differences, and are in part due to the cohort recruitment requirement that children survive to 6 weeks [25]. To ensure adequate representation of major ethnic groups in the study, the cohort is more ethnically diverse than national births [25].

Major data collection waves (DCWs) have occurred during late pregnancy, and when children were aged 9 months, 2 years, and 4.5 years. Information gathered at each DCW relate to six inter-connected domains of child development: health and wellbeing; cognitive and psychosocial; education; family and whānau (extended family); culture and identity; and neighbourhoods and societal context.

Children were included in the analyses only if their behaviour was measured at both ages 2 and 4.5 years. The final sample consisted of 5896 children (86% of the original sample). There were 348 children lost to follow up from the 2 year DCW to the 4.5 year DCW; however, 171 children who were not assessed at 2 years were followed up at 4.5 years. Children lost to follow up from age 2 to 4.5 years were more likely to have mothers who were younger, less educated and non-European, more likely to be part of an unplanned pregnancy, more likely to come from highly deprived areas at the 2 year DCW, and less likely to live in rural regions at age 2 (*ps* < .05). Further, children lost to follow up were also more likely to be categorised as abnormal on all SDQ scores at age 2 (*ps* < .05).

Children from the original, recruited sample that were not included in the current study were more likely to have mothers who were non-European, less educated and younger (*ps* < .001). Children not included were also more likely to be first born, part of an unplanned pregnancy, from an area of high deprivation, and from an urban area (*ps* < .05).

### Measures

#### Strengths and difficulties questionnaire

Behavioural difficulties were measured at 2 and 4.5 years using the mother-reported SDQ [26]. At 2 years, the preschool SDQ was used, while at 4.5 years the standard SDQ was administered. Each difficulties subscale and its corresponding items at ages 2 and 4.5 are provided in the Additional file 1: Table S1. Details of the minor differences between the preschool and standard SDQ are apparent in Table A1 and can also be found on the SDQ website [27]. The current study focuses on the difficulties subscales (emotional symptoms, peer problems, hyperactivity-inattention and conduct problems) as well as the total difficulties score.

Generally, each subscale is measured by five items, rated on a 3-point Likert scale as either *not true*, *somewhat true*, or *certainly true*. However, with the current study, an item (‘often fights with other children or bullies them’) corresponding to the conduct problems subscale was missing from the 4.5-year questionnaire (due to an administrative error); therefore, the subscale score was prorated to account for this missing item. Prorating was used to calculate scores for all subscales, though individuals were excluded if data was missing for more than two items for a subscale (or a single item in the case of the conduct problems subscale). The total difficulties score was calculated by summing the scores of the difficulties subscales.

We have previously found that the preschool SDQ shows generally acceptable psychometric properties at age 2 [3]. Consistent with our work on the SDQ at age 2 on structural validity, we found superior and acceptable model fit at age 4.5 years with a modified five-factor model that accounts for a positive construal method effect (*χ*2(237) = 3164.34; CFI = .926; TLI = .914; RMSEA = .046; for more information, see D’Souza et al. [3]). However, we found poor Cronbach’s alpha coefficients for both peer (α = .55) and conduct problems (α = .47). As estimates of Cronbach’s alpha can be affected by the number of scale items, it is possible that this low alpha for conduct problems is due to the reduced number of items [28, 29]. Cronbach’s alpha coefficients were acceptable for all other SDQ measures (α > .60).

SDQ subscales range from 0 to 10, and total difficulties ranges from 0 to 40. These scores were also categorised into normal, borderline and abnormal bands based on previously determined cut-offs [3, 26]. The abnormal band is typically used to identify children in need of further assessment and intervention, and is the method used by the B4 School Check to screen for children with social and emotional challenges [30–32]. SDQ measures were dichotomised into normal/borderline and abnormal in the current study, as we were primarily interested in movement into and out of the clinically significant abnormal range.

#### Sociodemographic and birth variables

Variables relating to the child or family’s social structure included mother’s ethnicity, mother’s education, mother’s age, child’s gender, parity, planned pregnancy, area-level deprivation, and rurality. Birthweight and gestational age were also of interest in the current study. Information on all variables except area-level deprivation and rurality were collected during the antenatal data collection wave. Information on area-level deprivation and rurality were collected during the 4.5 year DCW.

Mother’s self-prioritised ethnicity was categorised into four Level 1 Statistics New Zealand categories: European, Māori, Pacific, and Asian/Other [33]. If the individual identifies with multiple ethnicities, the self-prioritised ethnicity is what they consider to be their main ethnicity. In cases of mothers with multiple ethnic identifications who did not provide a self-prioritised ethnicity, external prioritisation was used. As utilised by Statistics New Zealand, external prioritisation gives precedence to responses in the following order: Māori, Pacific, Asian/Other, European [34].

Mother’s highest education was categorised into the following three levels: No secondary school; Secondary school/diploma/trade certificate; Bachelor’s degree or higher. Mother’s age during pregnancy was categorised as less than 20 years, 20–29 years, and 30 years and over. Area-level deprivation was measured using the NZDep2013, based on indicators of socioeconomic deprivation from the 2013 NZ census. Deprivation areas received a deprivation score from 1 (least deprived) to 10 (most deprived). Deprivation was categorised into high (deciles 8–10), medium (deciles 4–7), and low (deciles 1–3) deprivation.

### Data analysis

Correlations between SDQ measures at 2 and 4.5 years were calculated using Pearson correlation coefficients. Mean differences in SDQ scores were investigated using paired sample *t*-tests, with effect sizes calculated using Cohen’s *d* [35]. A contingency table was used to demonstrate the persistence and change in SDQ categorisation from 2 to 4.5 years.

A composite measure of behavioural stability was also created using the 2 year and 4.5 year SDQ total difficulties scores. Children were categorised as showing no difficulties (normal/borderline scores at 2 and 4.5 years), improved (abnormal score at 2 years only), later difficulties (abnormal score at 4.5 years only), and persistent difficulties (abnormal scores at 2 and 4.5 years). Chi-square analyses were used to evaluate the association between sociodemographic variables and behavioural stability, and to determine sociodemographic characteristics for each group. For continuous birth variables (i.e. birthweight, gestational age), ANOVAs were conducted. Due to the large number of bivariate analyses conducted, all *p*-values displayed have been adjusted for multiple comparisons using the Bonferroni correction.

## Results

### Correlation and differences in SDQ scores from 2 to 4.5 years

The correlation between SDQ measures at 2 and 4.5 years are presented in Table 1, as well as the *t*-value and effect size from the paired t-test comparing the mean scores at the two time points. Significant moderate correlations were found for all SDQ measures, Pearson *r *> 0.30, *ps* < .001. There were also significant differences in scores for all SDQ measures from 2 to 4.5 years, *ps* < .001. On average, all scores decreased from 2 to 4.5 years, except for emotional symptoms, which showed a negligible increase.

**Table 1:** Frequency distributions of behavioural categorisations, descriptive statistics, paired sample *t*-test results and correlation between age 2 and 4.5 year SDQ scores

	2 years			4.5 years			*t*	Cohen’s *d*	Pearson *r*
	Normal/ Borderline n (%)	Abnormal n (%)	M (SD)	Normal/ Borderline n (%)	Abnormal n (%)	M (SD)			
Total difficulties	5327 (90.5%)	558 (9.5%)	11.40 (5.11)	5222 (88.7%)	663 (11.3%)	9.86 (5.19)	25.15**	0.31	0.54**
Emotional symptoms	5497 (93.3%)	397 (6.7%)	1.80 (1.60)	5321 (90.3%)	573 (9.7%)	1.98 (1.77)	−6.71**	−0.10	0.43**
Peer problems	5330 (90.5%)	561 (9.5%)	2.17 (1.66)	5116 (86.8%)	775 (13.2%)	1.61 (1.60)	24.53**	0.31	0.37**
Hyperactivity-inattention	5428 (92.1%)	465 (7.9%)	4.32 (2.14)	5123 (86.9%)	770 (13.1%)	3.92 (2.26)	13.98**	0.17	0.45**
Conduct problems	5177 (87.8%)	716 (12.2%)	3.11 (1.97)	5237 (88.9%)	656 (11.1%)	2.35 (1.82)	27.57**	0.36	0.37**

*Note:* ** *p* < .001

### SDQ categorisations at 2 and 4.5 years

Table 1 also presents the normal/borderline and abnormal frequencies for each SDQ measure at ages 2 and 4.5 years. At age 2, abnormal total difficulties scores were observed for 9.5% of the cohort, 6.7% of children had abnormal scores for emotional symptoms, 9.5% had abnormal scores for peer problems, 7.9% had abnormal hyperactivity-inattention scores, and 12.2% had abnormal conduct problems. At 4.5 years, total difficulties were in the abnormal range for 11.3% of children. 9.7% of children had abnormal emotional symptoms, approximately 13% had abnormal scores for peer problems and hyperactivity-inattention, and 11.1% had abnormal conduct problems.

### Persistence and change in behaviour from 2 to 4.5 years

Table 2 presents the frequency distribution of behavioural categorisations for all SDQ measures cross-tabulated across ages 2 and 4.5 years. Of those who scored in the normal/borderline range at 2 years, approximately 90% remained in this range at 4.5 years (92%% total difficulties; 92.4% emotional symptoms; 89.1% peer problems; 89.1% hyperactivity-inattention; 91.1% conduct problems). A small percentage of children who scored in the normal/borderline range at 2 years showed an increase into the abnormal range at 4.5 years (8% total difficulties; 7.6% emotional symptoms; 10.9% peer problems; 10.9% hyperactivity-inattention; 8.9% conduct problems).

**Table 2:** Contingency table of 2 year and 4.5 year behavioural categorisations for each SDQ measure

4.5 years											
		Total difficulties		Emotional symptoms		Peer problems		Hyperactivity-Inattention		Conduct problems	
		Normal/ Borderline *n* (%)	Abnormal *n* (%)	Normal/ Borderline *n* (%)	Abnormal *n* (%)	Normal/ Borderline *n* (%)	Abnormal *n* (%)	Normal/ Borderline *n* (%)	Abnormal *n* (%)	Normal/ Borderline *n* (%)	Abnormal *n* (%)
2 years	Normal/Borderline	4899 (92.0%)	428 (8.0%)	5078 (92.4%)	419 (7.6%)	4748 (89.1%)	582 (10.9%)	4834 (89.1%)	594 (10.9%)	4718 (91.1%)	459 (8.9%)
	Abnormal	323 (57.9%)	235 (42.1%)	243 (61.2%)	154 (38.8%)	368 (65.6%)	193 (34.4%)	289 (62.2%)	176 (37.8%)	519 (72.5%)	197 (27.5%)

For children that scored in the abnormal range at 2 years, approximately 60–70% of children improved to score in the normal/borderline range for most SDQ measures (57.9% total difficulties; 61.2% emotional symptoms; 65.6% peer problems; 62.2% hyperactivity-inattention; 72.5% conduct problems). A notable percentage of children who scored in the abnormal range at 2 years showed persistence in abnormal scores at 4.5 years (42.1% total difficulties; 38.8% emotional symptoms; 34.4% peer problems; 37.8% hyperactivity-inattention; 27.5% conduct problems).

These results indicate four separate behavioural development profiles; children who showed no difficulties (i.e. remained in the normal/borderline range from 2 to 4.5 years), children who improved (i.e. moved from the abnormal range at 2 years to normal/borderline at 4.5 years), children who showed later difficulties (i.e. only showed abnormal scores at 4.5 years), and children who showed persistent difficulties (i.e. scored in the abnormal range at both 3 and 4.5 years). When looking at the proportions of each of these behavioural development profiles within the full study sample, approximately 80% of children showed no difficulties (83.2% total difficulties; 86.2% emotional symptoms; 80.6% peer problems; 82% hyperactivity-inattention; 80.1% conduct problems). Approximately 4–8% of the total cohort improved from 2 to 4.5 years (5.5% total difficulties; 4.1% emotional symptoms; 6.2% peer problems; 4.9% hyperactivity-inattention; 8.8% conduct problems). Of the total cohort, 7–10% showed later difficulties (7.3% total difficulties; 7.1% for emotional symptoms, 9.9% for peer problems; 10.1% for hyperactivity-inattention; 7.9% in conduct problems). Finally, approximately 3% of the overall cohort showed persistence in abnormal scores from 2 to 4.5 years (4% total difficulties; 2.6% emotional symptoms; 3.3% peer problems; 3% hyperactivity-inattention; 3.3% conduct problems).

### Association between behavioural stability, and sociodemographic and birth variables

Refer to Table 3 for results from the chi-square tests and for proportions discussed below. All sociodemographic variables were significantly associated with SDQ stability (*ps* < .05), except child’s gender and parity. Within the groups that showed behavioural difficulties during at least one time point (i.e. improved, later difficulties, and persistent difficulties), there was a greater proportion of children born to Māori or Pacific mothers relative to children showing no difficulties. Children with persistent difficulties had the greatest proportion of Māori and Pacific mothers.

**Table 3:** Association between behavioural stability profiles, and sociodemographic and birth variables

	Normal n (%) or M (SD)	Improved n (%) or M (SD)	Concurrent n (%) or M (SD)	Persistent n (%) or M (SD)	Χ2 or F-value
Mother’s ethnicity					
European	3055 (63.2)	99 (31.3)	148 (35.5)	44 (19.1)	586.5**
Maori	540 (11.2)	74 (23.4)	78 (18.7)	66 (28.7)	
Pacific	426 (8.8)	79 (25.0)	117 (28.1)	95 (41.3)	
Asian/Other	810 (16.8)	64 (20.3)	74 (17.7)	25 (10.9)	
Mother’s education					
No secondary school	214 (4.4)	36 (11.4)	53 (12.8)	44 (19.2)	356.90**
Secondary school/diploma/trade certificate	2399 (49.6)	222 (70.5)	282 (68.3)	157 (68.6)	
Bachelor’s degree or higher	2227 (46.0)	57 (18.1)	78 (18.9)	28 (12.2)	
Mother’s age					
< 20 years	139 (2.9)	28 (8.8)	35 (8.4)	31 (13.4)	291.21**
20 to 29 years	1652 (34.1)	167 (52.7)	220 (52.5)	134 (58.0)	
30 years and over	3053 (63.0)	122 (38.5)	164 (39.1)	66 (28.6)	
Child’s gender					
Male	2481 (50.6)	176 (54.5)	249 (58.2)	136 (57.9)	13.97
Female	2418 (49.4)	147 (45.5)	179 (41.8)	99 (42.1)	
Parity					
First born	2056 (42.5)	133 (42.1)	183 (43.9)	87 (37.8)	2.37
Subsequent birth	2785 (57.5)	183 (57.9)	234 (56.1)	143 (62.2)	
Planned pregnancy					
Yes	3207 (66.5)	140 (44.4)	191 (46.0)	80 (34.9)	198.37**
No	1619 (33.5)	175 (55.6)	224 (54.0)	149 (65.1)	
Area-level deprivation					
Low	1603 (34.4)	56 (18.5)	63 (15.3)	16 (7.2)	389.48**
Medium	1778 (38.2)	91 (30.1)	108 (26.2)	49 (22.2)	
High	1277 (27.4)	155 (51.3)	241 (58.5)	156 (70.6)	
Rurality					
Urban	4181 (89.8)	284 (94.0)	380 (92.2)	215 (97.3)	20.58*
Rural	477 (10.2)	18 (6.0)	32 (7.8)	6 (2.7)	
Gestational age in weeks	39.20 (1.71)	39.15 (1.67)	39.06 (1.67)	39.27 (1.42)	1.11
Birthweight in grams	3526.35 (553.65)	3497.95 (553.78)	3459.73 (540.71)	3510.15 (545.86)	2.07

Behavioural stability profiles were based on SDQ total difficulties categorisations at 2 and 4.5 years

Note: **p < .001, *p < .05

Relative to children with no difficulties, those who showed difficulties during at least one time point also had a greater proportion of mothers with no secondary school education, and fewer mothers who had achieved a Bachelor’s degree or higher. Children with no difficulties had the greatest proportion of mothers aged 30 years or over. In contrast, children who showed difficulties during at least one time point had a greater proportion of teen mothers relative to children showing no difficulties, with the persistent difficulties group showing the greatest proportion.

Relative to children showing no difficulties, the other groups had a greater percentage of children born from unplanned pregnancies (particularly those with persistent difficulties). Children within any of the groups showing difficulties during at least one time point had a notably greater percentage of children living in highly deprived areas, relative to those with no difficulties. Children with persistent difficulties in particular had the greatest proportion living in high deprivation areas relative to other groups. Those with persistent difficulties also had a greater proportion of children living in urban areas relative to children with no difficulties. The results from the ANOVAs showed that there was no significant difference between behavioural stability groups in either birthweight or gestational age (Table 3, *ps* > .05).

## Discussion

The current study was interested in evaluating the association between behaviour at two different time points in early childhood, and examining the persistence and change in the categorisation of behavioural difficulties during early childhood. We also examined the sociodemographic characteristics of the observed behavioural development profiles.

Consistent with our first hypothesis, we found that continuous measures of behaviour were moderately correlated and all scores except emotional symptoms showing a slight but significant decrease as the children got older. In contrast, emotional symptoms showed a slight but significant increase over time. The moderate correlation between behaviour at the two points is consistent with the work by Mathiesen and Sanson, who observed a correlation coefficient for total problems (*r* = 0.53) that is almost identical to the correlation coefficient for total difficulties observed in the current study. The decrease in externalising behaviour (conduct problems and hyperactivity-inattention), peer problems and total difficulties is also consistent with developmental changes associated with early childhood, and likely reflect the transient “terrible twos” [36, 37].

However, while these behaviours are likely normative and temporary for most children, we were also interested in children at the extreme end of these distributions in behaviour at both time points; that is, children who likely show serious behavioural problems, relative to other children of the same age. We were specifically interested in evaluating the rates of persistence and change for this categorisation of behavioural problems from ages 2 to 4.5 years. We observed that approximately 90% of those who scored in the normal/borderline range at 2 years remained within this range at 4.5 years. A small percentage of children who scored in the normal/borderline range at 2 years showed a later onset of behavioural problems by transitioning into the abnormal range at 4.5 years (7.7–11%). A notable percentage of children showed movement out of the abnormal range of behaviour at 4.5 years; 57.8% of children with abnormal scores improved their total difficulties by moving out of the abnormal range at 4.5 years. Similar percentages were found for most subscales (61–65.7%) except for conduct problems, where 72.6% of children with age 2 abnormal scores improved at 4.5 years. A higher percentage of improvement for the conduct problems subscale, relative to the other SDQ measures, is not surprising. Many of the behaviours measured by the conduct problems subscale (e.g. temper tantrums, disobedience) are behaviours that typically occur during early childhood and decrease in frequency with age [36, 37]. Therefore, this improvement in conduct problems from 2 to 4.5 years may simply reflect age-related changes in behaviour.

While it is encouraging to see that a substantial proportion of children showing serious early behavioural problems improved over the early childhood period, our results also indicate that many of the children displaying early behavioural problems persisted in these difficulties. Over 40% of children with abnormal total difficulties at age 2 continued to show abnormal total difficulties at 4.5 years, with slightly lower percentages observed for most subscales (27.4–39%). These percentages are similar to what has been reported in previous research. For example, Mathieson and Sanson found that 37% of children with behavioural problems at 18 months were also classified as having problems at 30 months [14] and Briggs-Gowan et al. found that 49.9% of children aged 12–40 months who initially showed total behaviour problems persisted in these behavioural problems 1 year later [15]. It is important to note that these children with persistent difficulties make up only approximately 3–4% of our total sample, though this is similar to the proportions of children with persistent difficulties in the studies mentioned above. Further, this proportion is to be expected, given that approximately 10% of children are categorised as showing serious behavioural difficulties at a single time point [3].

We also examined the association between preschool behavioural stability and sociodemographic factors. Our descriptive analyses indicated that, relative to children with no serious behavioural difficulties during early childhood, children who showed behavioural difficulties during at least one time point had a greater proportion whose mothers were younger, Māori and Pacific, and less educated, were more likely to live in highly deprived and urban areas, and were also more likely to be the result of an unplanned pregnancy. Those with persistent difficulties had a particularly higher proportion of the aforementioned characteristics relative to other groups. Teen parenting, lack of secondary school education and high area-level deprivation have previously been identified as risk factors for vulnerability in our cohort, with greater exposure to multiple risk factors being associated with poorer health outcomes from the immediate postnatal period through to 2 years [38].

NZ studies examining ethnic disparities in preschool behavioural problems or psychosocial wellbeing are lacking, though studies with adolescent NZ samples have reported that Māori and Pacific children were more likely than NZ European children to experience behavioural difficulties (Noel et al., 2013). However, it is important to acknowledge that these results are purely descriptive in nature, and we therefore cannot make claims about ethnic differences in behavioural difficulties based on the results of this study. The association between ethnicity and behavioural difficulties is likely to be complex; However, Gillies et al. (2014) have found that these ethnic disparities may be due to the influence of an accumulation of vulnerability risk factors earlier in life, including socioeconomic disadvantage and childhood trauma. In support of this, it was found that Māori and Pacific children within our cohort were more likely to be exposed to a greater number of antenatal vulnerability risk factors [38]. Therefore, the greater proportion of children with Māori and Pacific mothers within those showing behavioural difficulties is likely reflective of the greater exposure these ethnic groups have with socioeconomic disadvantage and early adversity. As such, results regarding ethnic differences should be interpreted with caution, and consider the broader social and historical factors likely contributing to these differences.

The results from the current study indicate that the persistence in early childhood behavioural problems observed in American and European samples are also apparent in a NZ cohort. These results support the need for repeated screening for behavioural problems, beginning from as early as 2 years. This could be applied by including the SDQ in the earlier health and development checks conducted in NZ, such as the 2–3 year check that is conducted just prior to the B4 School Check [20]. This would enable even earlier intervention or at least the identification of children who are showing persistent difficulties at both the 2–3 year check and the B4 School Check. To inform any intervention efforts, future research should investigate family and environmental factors influencing the persistence and change in early childhood behavioural problems.

It is important to note that the SDQ is appropriate as a screening instrument for behavioural difficulties, and not as a diagnostic tool. We were unable to evaluate the sensitivity or specificity of the clinical cut-offs used in this study against more formal clinical diagnoses. While we had some diagnostic information at age 2 years, very few children had received any diagnoses at this age and we do not have this information at 4.5 years. However, we may be able to investigate this in future by linking with administrative health records.

The current study was also somewhat limited in its investigation of persistence and change in childhood behavioural difficulties, as we could only track the stability in behavioural problems over two DCWs. As such, change across two time points may also be due to measurement error, rather than true change. However, *Growing Up in New Zealand* is currently collecting data for its 8 year DCW, which includes the SDQ. Future investigation will examine the trajectories of behavioural difficulties across the 2 year, 4.5 year and 8 year DCWs, which will provide us more insight into the stability of childhood behavioural difficulties. The work from this study will be useful in informing this future research.

An additional limitation was the reduction in the representativeness of the sample as a result of attrition. Compared to the broadly generalisable original sample recruited by *Growing Up in New Zealand*, the children in the current study were more likely to have mothers who were European, more educated and older, more likely to come from less deprived areas and less likely to come from urban areas. However, while there is a statistically significant difference in the sociodemographic characteristics of children included and not included in the current study, the study’s analytic sample still showed considerable diversity on these key sociodemographic measures and is therefore still an important and relevant resource.

## Conclusions

Our results ultimately show that the majority of children who present with abnormal behavioural scores at age 2 typically improved by 4.5 years. However, there was still a significant proportion of children with an abnormal categorisation at age 2 who persisted in their difficulties at 4.5 years. There was also a small percentage of children who initially did not show behavioural problems but were classified as having abnormal scores at 4.5 years. Further, each of these groups, but particularly those with persistent difficulties, had a larger proportion of children experiencing risk factors for vulnerability relative to children with no difficulties. This study was intended to be descriptive in nature, and therefore does not address the complex associations between preschool behavioural stability and sociodemographic factors, though *Growing Up in New Zealand* aims to address this in future studies. Future research will also aim to identify proximal and distal family and environmental factors that may contribute to this persistence or change in problem behaviours. Nevertheless, findings from the current study are novel, given that, to our knowledge, we are the first to utilise the SDQ during multiple time points in the preschool period. Importantly, as our results indicate that some, but not all, children who show serious behavioural difficulties continue to persist in these difficulties across early childhood, repeated screening for behavioural problems is important.

## Additional file

Additional file 1: **Table S1.** SDQ difficulties subscales and the corresponding items at ages 2 and 4.5 years. Table displaying each SDQ difficulties subscale and its corresponding items at ages 2 and 4.5, to demonstrate the difference between the preschool and standard versions of the SDQ. (DOCX 12 kb)

## Data Availability

The data that support the findings of this study are available from *Growing Up in New Zealand* but restrictions apply to the availability of these data, which were used under license for the current study, and so are not publicly available. *Growing Up in New Zealand* data can be made available to external researchers with permission from the study’s Data Access Committee, providing that the Data Access Protocol is followed. Details of this can be found on the study’s website www.growingup.co.nz.

## Abbreviations

DCW: Data Collection Wave
NZ: New Zealand
SDQ: Strengths and Difficulties Questionnaire
