# Supplementary material for: Persistence and change in behavioural problems during early childhood
Source: BMC Pediatr. 2019 Jul 26;19:259. doi: 10.1186/s12887-019-1631-3 (PMC6659228; doi:10.1186/s12887-019-1631-3)
Supplement: Supplementary file 1 — Table S1. SDQ difficulties subscales and the corresponding items at ages 2 and 4.5 years. Table displaying each SDQ difficulties subscale and its corresponding items at ages 2 and 4.5, to demonstrate the difference between the preschool and standard versions of the SDQ. (DOCX 12 kb) [file 12887_2019_1631_MOESM1_ESM.docx]

| Table S1.  SDQ difficulties subscales and the corresponding items at ages 2 and 4.5 years. | | |
| --- | --- | --- |
| **SDQ measure** | **2 year items** | **4.5 year items** |
| Emotional symptoms | Often complains of headaches, stomach-aches, or sickness | Often complains of headaches, stomach-aches, or sickness |
|  | Many worries, often seems worried | Many worries or often seems worried |
|  | Often unhappy, down-hearted or tearful | Often unhappy, down-hearted or tearful |
|  | Nervous or clingy in new situations, easily loses confidence | Nervous or clingy in new situations, easily loses confidence |
|  | Many fears, easily scared | Many fears, easily scared |
| Peer problems | Rather solitary, tends to play alone | Rather solitary, prefers to play alone |
|  | Has at least one good friend* | Has at least one good friend* |
|  | Generally liked by other children* | Generally liked by other children* |
|  | Picked on or bullied by other children | Picked on or bullied by other children |
|  | Gets on better with adults than with other children | Gets on better with adults than with other children |
| Hyperactivity-Inattention | Restless, overactive, cannot stay still for long | Restless, overactive, cannot stay still for long |
|  | Constantly fidgeting or squirming | Constantly fidgeting or squirming |
|  | Easily distracted, concentration wanders | Easily distracted, concentration wanders |
|  | Can stop and think things out before acting* | Thinks things out before acting* |
|  | Sees tasks through to the end, good attention span* | Good attention span, sees chores or work through to the end* |
| Conduct problems | Often has temper tantrums or hot tempers | Often loses temper |
|  | Generally obedient, usually does what adults request* | Generally well behaved, usually does what adults request* |
|  | Often fights with other children or bullies them |  |
|  | Often argumentative with adults | Often lies or cheats |
|  | Can be spiteful to others | Steals from home, pre-school or elsewhere |
| *Note:* There are five additional items corresponding to the SDQ prosocial behaviour subscale that are not displayed here, as this measure was not of interest in the current study.  * reverse-score items. | | |
